# Associations Between Glycemic Control, Self-Reported Gingival Bleeding and Lifestyle Factors in Hospitalized Diabetic Patients

**DOI:** 10.3390/jcm14124201

**Published:** 2025-06-13

**Authors:** Aida Meto, Adora Shpati, Adela Alushi, Tommaso Filippini, Alba Kamberi, Agron Meto, Adem Alushi

**Affiliations:** 1 Department of Dentistry, Faculty of Dental Sciences, University of Aldent, 1007 Tirana, Albania; adorashpati@yahoo.com (A.S.); adela.alushi@ual.edu.al (A.A.); albakamberi@hotmail.com (A.K.); agron.meto@ual.edu.al (A.M.); 2 Department of Surgery, Medicine, Dentistry and Morphological Sciences with Interest in Transplant, Oncology and Regenerative Medicine, University of Modena and Reggio Emilia, 41125 Modena, Italy; 3 Department of Dental Research Cell, Dr. D.Y. Patil Dental College and Hospital, Dr. D.Y. Patil Vidyapeeth, Pimpri, Pune 411018, Maharashtra, India; 4 Department of Biomedical, Metabolic and Neural Sciences, University of Modena and Reggio Emilia, 41125 Modena, Italy; 5 Department of Dental Therapy, Faculty of Dental Medicine, University of Medicine, 1005 Tirana, Albania; adem.alushi@umed.edu.al

**Keywords:** diabetes mellitus, HbA1c levels, Quantitative Gingival Bleeding Index, gingival health, oral health, dental care, hospitalized patients

## Abstract

**Background/Objectives:** Diabetes mellitus is associated with significant health complications, including challenges in periodontal health. Gingival inflammation is especially common among diabetic patients and can significantly impact overall diabetes management. This study aims to investigate the correlation between glycosylated hemoglobin (HbA1c) levels, an established marker of glycemic control, and gingival inflammation using the Quantitative Gingival Bleeding Index (QGBI) among hospitalized diabetic patients, thereby placing the question within a broader clinical context. **Methods**: The study enrolled 671 hospitalized patients with diabetes complications at “Mother Theresa University Hospital” in Tirana, AL, USA. Glycemic control was assessed through glycosylated hemoglobin (HbA1c) levels, and gingival health was evaluated using the Quantitative Gingival Bleeding Index (QGBI). Behavioral variables were also documented, including smoking habits and routine oral care practices. Spearman’s correlation coefficient (r_s_) was applied to determine the relationship between HbA1c levels and QGBI scores. **Results**: Our findings revealed a strong positive correlation between glycemic control (HbA1c) and gingival inflammation (QGBI) among the participants (Spearman’s coefficient r_s_ = 0.868, *p* < 0.001). Additionally, significant positive associations were observed between behavioral factors, such as smoking habits and regular oral care practices, further underscoring their role in periodontal health in diabetic patients. **Conclusions**: The study highlights a significant positive relationship between poor glycemic control and increased gingival inflammation, emphasizing the importance of periodontal health in the comprehensive management of diabetes mellitus. Our results support integrating periodontal evaluation and management into standard diabetes care, which could improve patient outcomes and overall well-being.

## 1. Introduction

**Figure 1:**
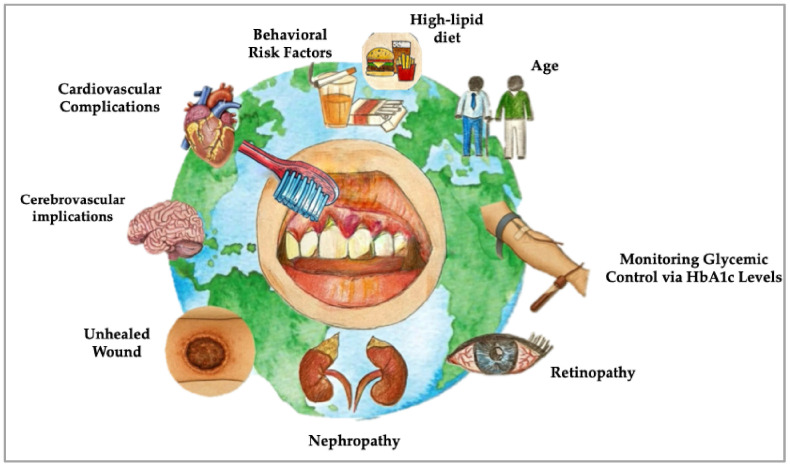
This illustration depicts the multisystemic impact of periodontal inflammation in individuals with diabetes mellitus. A clinical representation of gingival bleeding and inflammation at the center highlights active periodontal disease. Around the central illustration are depicted several complications associated with diabetes, along with key contributing factors. Cardiovascular and cerebrovascular implications are shown, reflecting the increased risk of heart disease and stroke associated with chronic inflammation and poor glycemic control. An unhealed wound reflects the reduced healing capacity often seen in diabetes. Nephropathy and retinopathy represent microvascular damage affecting the kidneys and eyes, respectively. A blood draw highlights the importance of monitoring glycemic control through HbA1c levels. Behavioral risk factors such as smoking and alcohol use, a high-lipid diet, and aging are identified as contributors to disease progression. A world map in the background underscores the global relevance of these interrelated health challenges and the potential of integrating oral health assessments into comprehensive diabetes care. Illustration by Nensi Kallfani.

Diabetes mellitus is a long-term metabolic condition characterized by persistent hyperglycemia due to reduced insulin production, insulin resistance, or both. It affects millions worldwide and is a public health concern, contributing to significant morbidity and mortality [1]. Type 1 diabetes is caused by the autoimmune destruction of pancreatic β-cells and requires lifelong insulin therapy. Type 2 diabetes, on the other hand, is mainly linked to insulin resistance and progressive β-cell dysfunction, often associated with lifestyle factors like obesity, high-fat diets, and physical inactivity [2,3,4]. As illustrated in Figure 1, periodontal inflammation in diabetic patients is interconnected with systemic complications, including cardiovascular, renal, and ocular effects, and is influenced by both behavioral and biological risk factors.

Glycated hemoglobin (HbA1c) is a widely recognized biomarker for long-term glycemic control, reflecting average blood glucose levels over the past two to three months [5,6]. Higher HbA1c levels are associated with an increased risk of both microvascular (retinopathy, nephropathy, and neuropathy) and macrovascular (cardiovascular disease, stroke, and peripheral artery disease) complications [7,8,9,10]. The growing prevalence of diabetes, projected to increase from 536.6 million in 2021 to nearly 783.2 million by 2045, highlights the urgent need for more effective management strategies to reduce associated complications [11,12].

One often-overlooked complication of diabetes is its impact on gingival health. Diabetes and periodontal disease have a well-documented bidirectional relationship, where poor glycemic control worsens periodontal inflammation and untreated periodontal disease further impairs glucose regulation [13,14]. Chronic hyperglycemia contributes to increased inflammation and impaired wound healing, making individuals with diabetes more susceptible to gingival bleeding and periodontitis [15,16].

The Quantitative Gingival Bleeding Index (QGBI) objectively measures gingival inflammation by quantifying bleeding during brushing [17]. Compared to traditional bleeding indices that only show whether bleeding is present or not, QGBI provides a graded measure of bleeding severity, giving better insight into the progression of periodontal disease [18,19]. Given the increased susceptibility of diabetic patients to oral inflammation, QGBI may serve as an important tool for monitoring gingival health and its potential impact on glycemic control [20,21,22]. In Albania, patients are not regularly monitored through laboratory examinations, often due to the associated costs. To address this gap, we considered evaluating an index (QGBI) that patients can assess at home, enabling them to recognize early warning signs and obtain timely consultation with a specialist.

This study aims to investigate the correlation between HbA1c levels and the QGBI in hospitalized diabetic patients, providing insight into the relationship between glycemic control and gingival health. Furthermore, we examined the impact of lifestyle factors, including smoking habits and the frequency of dental check-ups, on gingival bleeding severity. By understanding these associations, our findings may contribute to a more integrated diabetes management approach, emphasizing both systemic and oral health.

## 2. Methods

### 2.1. Study Population

This cross-sectional study enrolled patients with diabetes at Mother Teresa University Hospital in Tirana, AL, USA, between May and December 2023. This care facility serves a diverse patient population from various regions of Albania, requiring specialized services provided exclusively by the country’s sole tertiary health center. The center delivers advanced, multidisciplinary care aimed at addressing major complications linked to diabetes. The total number of patients participating in our study was 671, which included 388 (57.8%) male patients and 283 (42.2%) female patients.

Inclusion Criteria: Hospitalized patients aged 16 to 85 years with a diagnosis of type 1 or type 2 diabetes mellitus were included, as individuals in this age range can manage their condition and provide reliable self-reported information.

Exclusion Criteria: Participants with thyroid disorders were excluded due to their potential impact on HbA1c levels, which could confound the study’s assessment of diabetes-related glycemic control. Similarly, individuals with anemia or chronic renal failure, altered liver function, and splenomegaly were excluded, as both conditions may influence HbA1c test results. To isolate the effects of smoking on periodontal health and diabetes management, participants with a history of alcohol consumption were also excluded. Additional exclusions were patients with other metabolic or systemic conditions, those on medication affecting gingival bleeding, individuals with peripheral diabetic neuropathy, edentulous patients unable to undergo QGBI assessment, and those who declined to participate. Participants were given clear, standardized instructions before data collection to ensure consistent and reliable QGBI measurements. All assessments followed a validated protocol to keep patient-reported bleeding severity data uniform. These well-defined procedures helped recruit a similar group of participants, improving the accuracy and reliability of the study results.

### 2.2. Data Collection

Medical records were reviewed to determine each patient’s type of diabetes, and HbA1c levels were measured using standardized laboratory tests. We conducted a structured interview to collect detailed information on participants’ behavioral patterns, including smoking habits, oral care habits, hypertension, and awareness of the connection between diabetes and gingival health. Gingival health was assessed using the QGBI, a validated tool for evaluating gingival bleeding based on patient-reported outcomes through a structured questionnaire. Rather than a simple self-assessment, patients reported the severity of their gingival bleeding, which the interviewer interpreted and scored according to the QGBI criteria. Patients were asked to describe their bleeding experience during toothbrushing, and their responses were categorized and scored based on the QGBI criteria outlined in Table 1.

### 2.3. Statistical Analysis

The data was initially coded in MS Excel, double-checked for accuracy, and subsequently imported into Stata software (StataNow/MP 18.5, StataCorp LCC, College Station, TX, USA, 2023). The Shapiro–Wilk test was used to evaluate the data’s conformity to a normal distribution. Descriptive statistics were reported, including absolute and relative frequencies, as well as median and interquartile range (IQR) for continuous variables that showed a non-normal distribution. The relationship between glycemic control (measured by HbA1c levels) and gingival bleeding severity (measured by QGBI) was assessed using Spearman’s correlation coefficient (r_s_).

We used multivariable linear regression analysis to examine the associations between patients’ characteristics and QGBI scores, adjusting for all relevant factors and potential confounders, including HbA1c levels, sex, age, smoking status, hypertension, oral care habits, and awareness of the diabetes gingival health link. Since age and HbA1c were non-normally distributed, they were log-transformed to perform the analysis. Results were reported as linear regression coefficients (r_c_) with 95% confidence intervals (CI). We also evaluated the risk of moderate to severe gingival bleeding (QGBI scores ≥ 2) based on patient characteristics by calculating odds ratios (OR) with corresponding 95% confidence intervals (CI).

### 2.4. Ethics

Ethical approval for the study (Protocol no. 144/23) was obtained from the Institutional Review Board of the University of Aldent before data collection. Informed consent was secured from all participants, ensuring their voluntary participation. Appropriate measures were taken to ensure participant confidentiality and anonymity, maintaining the highest standards of privacy. No identifying information was recorded through the questionnaires, and responses were securely stored in a password-protected file. To prevent identification through indirect identifiers or combined information, additional precautions were implemented to keep all patient data secure and fully de-identified throughout the research process.

## 3. Results

Patient characteristics are presented in Table 2. A total of 671 patients (388 males and 283 females) were enrolled, with a median age of 56 years (IQR: 31–79). Smokers and non-smokers were nearly equally represented in the study population. Type 2 diabetes was the most frequent diagnosis, with a median HbA1c of 7.9% (IQR: 7.1–9.4). The majority of patients (73.2%) did not attend regular oral check-ups, while 17.3% reported annual visits and 9.5% visited the dentist twice a year.

The awareness of the link between diabetes and gingival health was generally very low, with only about 2% of patients reporting knowledge of this relationship. QGBI scores indicated mild to severe gingival bleeding in the vast majority of patients.

The analysis showed a strong association between smoking and poor oral care habits, with smokers being more likely than non-smokers to skip regular dental check-ups (Table 3).

The distribution of patient characteristics by QGBI scores showed a positive association with age (Table 4). An increasing proportion of smokers was also observed with higher QGBI scores, particularly in cases of moderate to severe gingival bleeding.

Patients who did not attend routine oral check-ups had higher QGBI scores. In contrast, awareness of the link between diabetes and gingival health was more common among individuals without signs of inflammation or bleeding (QGBI = 0), though this finding is based on a limited number of cases.

**Figure 2:**
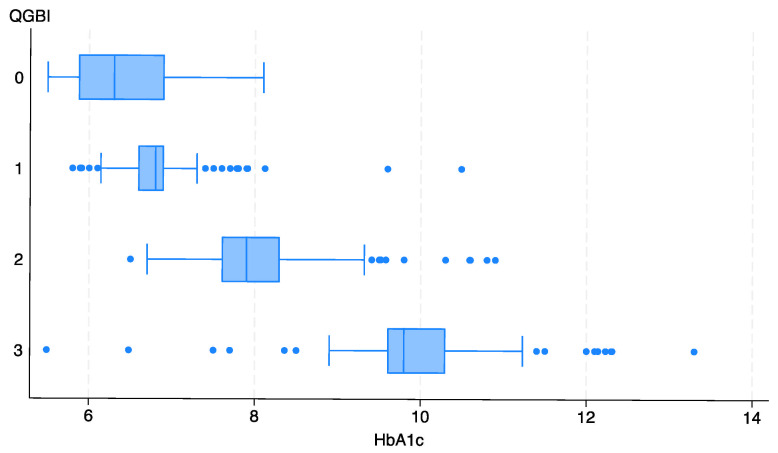
Box plot showing the relationship between HbA1c levels and QGBI scores, highlighting the trend of increasing glycemic levels with greater gingival bleeding severity.

Regarding diabetes type, patients with type 2 generally showed higher levels of gingival bleeding compared to those with type 1. Similarly, a higher prevalence of individuals with poorly controlled HbA1c levels (≥7.0%) was observed among those with higher QGBI scores. This trend is further supported by the distribution of HbA1c levels across QGBI categories, as shown in Figure 2.

Spearman’s correlation analysis revealed a strong positive association between HbA1c levels and QGBI scores (r_s_ = 0.868, 95% CI: 0.848–0.885).

Multivariate linear regression analysis showed a positive association between QGBI levels, patient age (r_c_ = 0.247, 95% CI: 0.180–0.314), smoking (r_c_ = 0.121, 95% CI: 0.057–0.184), and HbA1c levels (r_c_ = 3.362, 95% CI: 3.145–3.580) (Table 5). A negative association was observed with sex (r_c_ = −0.211, 95% CI: −0.275 to −0.147) and, to a limited extent, with regular oral care (r_c_ = −0.071, 95% CI: −0.145 to 0.003). No significant associations were found for hypertension (r_c_ = −0.015, 95% CI: −0.074 to 0.043) or awareness of the link between diabetes and gingival health (r_c_ = −0.080, 95% CI: −0.282 to 0.123).

Logistic regression analysis for moderate to severe gingival bleeding (QGBI ≥ 2) showed a higher risk associated with increasing age (OR = 1.05, 95% CI: 1.03–1.07), smoking (OR = 4.23, 95% CI: 1.96–9.14), and elevated HbA1c levels (OR = 20.97, 95% CI: 11.10–39.62). In contrast, female patients had a significantly lower risk (OR = 0.13, 95% CI: 0.06–0.26) (Table 5). Routine dental check-ups were linked to a slightly reduced risk, while awareness of the link between diabetes and gingival health showed a stronger protective effect.

## 4. Discussion

Our study highlights a significant correlation between HbA1c levels and QGBI scores in hospitalized diabetic patients, suggesting that gingival health may serve as a simple and accessible indicator of glycemic control. This aligns with existing research on the bidirectional relationship between diabetes and periodontal disease, in which systemic inflammation, driven by hyperglycemia and the accumulation of advanced glycation end products (AGEs), worsens both conditions [13,23,24]. Oral health problems are recognized as potential complications of diabetes, with evidence suggesting they may adversely affect metabolic control [25,26].

While QGBI is a practical and patient-friendly tool for assessing gingival bleeding, it does not account for deeper periodontal conditions, such as clinical attachment loss and probing depth. Our study’s lack of a plaque index limits a comprehensive assessment of gingival inflammation, as plaque accumulation plays a crucial role in gingival bleeding and periodontal disease progression [27,28]. Subsequent studies should incorporate plaque indices and inflammatory biomarkers to better understand the link between periodontal health and glycemic control.

Our findings show a significant and somewhat unexpected positive association between smoking and QGBI. Although smoking is traditionally associated with vasoconstriction and reduced clinical signs of gingival bleeding, recent evidence, including our results, suggests that smoking may not always suppress visible inflammation as previously believed. Instead, in certain populations, it may enhance inflammatory responses, contributing to elevated bleeding indices. This aligns with existing research showing that smoking impairs immune function, increases oxidative stress, and accelerates the destruction of periodontal tissues [29,30]. Moreover, population-based studies conducted in three South American cities reinforce the link between smoking and increased gingival inflammation across diverse groups, suggesting that the effect may vary depending on exposure level, duration, and individual susceptibility [30]. Among individuals with diabetes, smoking has an even greater impact, being strongly associated with poor metabolic control, which may further worsen periodontal inflammation and bleeding [30,31]. Because of these complex interactions, the following studies should broaden the focus beyond tobacco use to consider other lifestyle factors, such as dietary habits, physical activity, psychosocial stress, and examine their combined effects on periodontal health in diabetic populations. This approach could help identify important behavioral and metabolic factors involved in periodontal disease and support the development of more effective, coordinated prevention strategies across both dental and medical practice.

Hypertension, a common comorbidity in diabetes, is a known contributor to gingival inflammation. It is well established that hypertension induces systemic inflammation, which may worsen periodontal conditions [32]. Additionally, certain antihypertensive medications, particularly calcium channel blockers, are linked to gingival hyperplasia, which complicates oral hygiene and increases the risk of gingival bleeding [33,34]. Other classes of antihypertensive drugs may also reduce salivary flow, further elevating the risk of periodontal disease [35,36].

However, hypertension was not included as a confounding variable in our study. Future research should stratify patients based on hypertension status and medication use to better understand their impact on gingival health.

Age was also a significant factor influencing gingival health, with older patients showing higher QGBI scores. This aligns with findings that aging is linked to declining periodontal health due to cumulative plaque exposure, weakened immune response, and slower healing [37,38]. These findings emphasize the importance of targeted education and preventive strategies for older diabetic patients, especially in promoting oral hygiene and regular dental visits.

In addition to age, our study highlights the influence of modifiable and non-modifiable risk factors on periodontal health. Factors such as smoking, oral hygiene habits, sex, and diabetes type were all associated with gingival inflammation. Notably, males showed greater susceptibility, which is consistent with previous studies showing sex-based differences in periodontal disease progression due to hormonal, immune, and behavioral factors [39,40]. Furthermore, research suggests that individuals with type 1 diabetes tend to have better periodontal health outcomes compared to those with type 2, likely due to differences in metabolic control and treatment approaches [39].

What makes this study novel is the use of QGBI as a skilled self-assessment tool, where patients, after being shown how to recognize different levels of gingival bleeding, reported their symptoms. This approach demonstrates the potential of QGBI as a cost-effective and accessible method for the early identification of poor glycemic control, without requiring direct dental examination. Its correlation with HbA1c enables patients to recognize that an increase in gingival bleeding (QGBI) may indicate poor diabetes management and elevated HbA1c levels. This highlights its potential role in diabetes self-monitoring, empowering patients to obtain timely medical or dental intervention when necessary.

However, our study also found a general lack of awareness among diabetic patients regarding the link between periodontal health and glycemic control. Previous research shows that better oral health awareness is associated with better gingival health outcomes in diabetes patients [41,42]. This underscores the urgent need for integrated education programs within healthcare systems that emphasize the importance of oral health in diabetes management.

A main limitation of this study is the absence of detailed clinical data, like probing depth and attachment loss, which are critical for a comprehensive evaluation of periodontal health. Due to difficult hospital conditions and patients not agreeing to visit our university dental clinic for a periodontal check-up, we could not perform a more detailed clinical evaluation. Additionally, relying on self-reported QGBI may introduce potential bias, even though standardized instructions were given. While earlier research suggests that self-reported gingival bleeding can serve as a useful screening tool [22], future studies should combine it with clinical measures to enhance accuracy.

Future research should use longitudinal study designs to investigate further how glycemic control, periodontal health, and lifestyle factors, particularly dietary habits, interact and influence both diabetes management and gingival health [43]. It would also be valuable to perform subgroup analyses based on factors such as obesity, smoking, hypertension, and the use of antihypertensive medications to better understand their impact on gingival bleeding.

## 5. Conclusions

This study highlights the link between HbA1c levels and QGBI in hospitalized diabetes patients, reinforcing the importance of gingival health in diabetes management. A key finding was the lack of awareness of this connection, pointing to the need for better patient education. Additionally, the positive link between smoking habits and regular dental visits suggests that informed patients are more likely to prioritize oral health.

Integrating structured educational programs, routine dental examinations, and interdisciplinary collaboration between medical and dental professionals is essential for enhancing glycemic control and periodontal health. These preventive measures can increase patient awareness, encourage healthier behaviors, and reduce periodontal complications in individuals with diabetes.

## Figures and Tables

**Table 1:** QGBI scores.

Score	Description
0	No bleeding during brushing, with bristles completely free of blood stains.
1	Slight bleeding during brushing, with blood visible only on the bristle tips.
2	Moderate bleeding during brushing, with blood staining about half the length of the toothbrush bristles from the tip downward.
3	Severe bleeding during brushing, with blood covering the full length of all bristles and the brush head.

**Table 2:** Characteristics of study participants (n = 671). Values are reported as numbers (N) and percentages (%) unless otherwise specified. IQR: interquartile range.

Characteristics	N (%)
**Sex**	
Male	388 (57.8)
Female	283 (42.2)
**Age**	
Median (IQR)	56 (31–79)
<18 years	15 (2.2)
18–30 years	148 (22.1)
31–64 years	235 (35.0)
65–79 years	128 (19.1)
≥80 years	145 (21.6)
**Diabetes type**	
Type 1	89 (13.3)
Type 2	582 (86.7)
**HbA1c**	
Median (IQR)	7.9 (7.1–9.4)
HbA1c ≤ 7.0%	160 (23.8)
HbA1c > 7.0%	512 (76.2)
**Hypertension**	
Patients with hypertension	256 (38.2)
Patients without hypertension	415 (61.8)
**Smoking habits**	
Non-smokers	320 (47.7)
Smokers	351 (52.3)
**Routine oral check-up**	
Not regularly	491 (73.2)
Once a year	116 (17.3)
Twice a year	64 (9.5)
**Awareness of the relation between diabetes and gingival health**	
No	657 (97.9)
Yes	14 (2.1)
**Quantitative Gingival Bleeding Index**	
Normal (Score = 0)	16 (2.4)
Mild inflammation with no bleeding (Score = 1)	184 (27.4)
Moderate inflammation and bleeding on probing (Score = 2)	285 (42.5)
Severe inflammation and tendency of spontaneous bleeding (Score = 3)	186 (27.7)

**Table 3:** Relation between routine oral care frequency and smoking habits.

	Non-Regularly	Once a Year	Twice a Year
	N (%)	N (%)	N (%)
**Smoking habits**			
Non-Smokers	179 (36.5)	86 (74.1)	55 (85.9)
Smokers	312 (63.5)	30 (25.9)	9 (14.1)

**Table 4:** Distribution of patients’ characteristics according to QGBI scores.

	QGBI = 0	QGBI = 1	QGBI = 2	QGBI = 3
**Age categories**				
<18 years	0 (0.0)	12 (6.5)	3 (1.1)	0 (0)
18–30 years	6 (37.5)	84 (45.7)	53 (18.6)	5 (2.7)
31–64 years	9 (56.3)	75 (40.8)	137 (48.1)	14 (7.5)
65–79 years	1 (6.2)	10 (5.4)	46 (16.1)	71 (38.2)
≥80 years	0 (0)	3 (1.6)	46 (16.1)	96 (51.6)
**Smoking habits**				
Non-smokers	12 (75.0)	150 (81.5)	102 (35.8)	56 (30.1)
Smokers	4 (25.0)	34 (18.5)	183 (64.2)	130 (69.9)
**Hypertension**				
No	11 (68.8)	119 (64.8)	171 (60.0)	114 (61.3)
Yes	5 (31.2)	65 (35.3)	114 (40.0)	72 (38.7)
**Routine oral check-up**				
Not regularly	4 (25.0)	99 (53.8)	215 (75.4)	173 (93.0)
Once a year	5 (31.3)	47 (25.5)	53 (18.6)	11 (5.9)
Twice a year	7 (43.7)	38 (20.6)	17 (6.0)	2 (1.1)
**Awareness**				
No	14 (87.5)	180 (97.8)	277 (97.2)	186 (100)
Yes	2 (12.5)	4 (2.2)	8 (2.8)	0 (0.0)
**Diabetes**				
Type 1	4 (25.0)	58 (31.5)	25 (8.8)	2 (1.1)
Type 2	12 (75.0)	126 (68.5)	260 (91.2)	184 (98.9)
**HbA1c**				
HbA1c ≤ 7.0%	12 (75.0)	140 (76.1)	5 (1.8)	2 (1.1)
HbA1c > 7.0%	4 (25.0)	44 (23.9)	280 (98.2)	184 (98.9)

**Table 5:** Results from linear regression and logistic models, respectively, between continuous QGBI scores and risk of moderate/severe gingival bleeding (QGBI ≥ 2) and patients’ characteristics. Regression coefficient (r_c_), odds ratio (OR), and 95% confidence interval (CI).

Characteristics	r_c_	(95% CI)	OR	(95% CI)
HbA1c (1-unit increase)	3.362	(3.145; 3.58)	20.97	(11.10–39.62)
Age (1-year increase)	0.247	(0.18; 0.314)	1.05	(1.03–1.07)
Sex (females)	−0.211	(−0.275; −0.147)	0.13	(0.06–0.26)
Smokers	0.121	(0.057; 0.184)	4.23	(1.96–9.14)
Hypertension	−0.015	(−0.074; 0.043)	1.18	(0.57–2.44)
Regular oral care	−0.071	(−0.146; 0.003)	0.97	(0.43–2.19)
Awareness	−0.08	(−0.282; 0.123)	0.46	(0.05–4.36)

## Data Availability

The data presented in this study are available on request from the corresponding authors.
